# Multivariate Analysis of Bivariate Phase-Amplitude Coupling in EEG Data Using Tensor Robust PCA

**DOI:** 10.1109/TNSRE.2021.3092890

**Published:** 2021-07-12

**Authors:** Tamanna T. K. Munia, Selin Aviyente

**Affiliations:** Department of Electrical and Computer Engineering, Michigan State University, East Lansing, MI 48824 USA

**Keywords:** Phase amplitude coupling, time frequency distribution, multivariate, tensor decomposition, HoRPCA, EEG

## Abstract

Cross-frequency coupling is emerging as a crucial mechanism that coordinates the integration of spectrally and spatially distributed neuronal oscillations. Recently, phase-amplitude coupling, a form of cross-frequency coupling, where the phase of a slow oscillation modulates the amplitude of a fast oscillation, has gained attention. Existing phase-amplitude coupling measures are mostly confined to either coupling within a region or between pairs of brain regions. Given the availability of multi-channel electroencephalography recordings, a multivariate analysis of phase amplitude coupling is needed to accurately quantify the coupling across multiple frequencies and brain regions. In the present work, we propose a tensor based approach, i.e., higher order robust principal component analysis, to identify response-evoked phase-amplitude coupling across multiple frequency bands and brain regions. Our experiments on both simulated and electroencephalography data demonstrate that the proposed multivariate phase-amplitude coupling method can capture the spatial and spectral dynamics of phase-amplitude coupling more accurately compared to existing methods. Accordingly, we posit that the proposed higher order robust principal component analysis based approach filters out the background phase-amplitude coupling activity and predominantly captures the event-related phase-amplitude coupling dynamics to provide insight into the spatially distributed brain networks across different frequency bands.

## I. Introduction

NEURONAL oscillations and the interactions between them are believed to play a key role in understanding the cognitive function of the brain [1], [2]. The interaction and coordination between oscillations at different frequencies is defined as cross-frequency coupling (CFC) [3], [4]. CFC plays a fundamental role in large scale neuronal encoding and communication by providing temporal and spatial dynamics necessary for routing information through brain networks [3], [5]. CFC is an umbrella term to define a variety of phenomena like phase-phase coupling (PPC), amplitude-amplitude coupling (AAC), and phase-amplitude coupling (PAC).

One of the most studied forms of CFC is PAC, which quantifies the interplay between the phase of a slower oscillation and the envelope of a faster oscillation [4]. PAC has been reported to play a critical role in the execution of cognitive functions [6], attention selection [7], long-term memory processing [8], and sensory signal detection [9]. Although a variety of metrics have been developed to quantify PAC, most of these measures are bivariate in nature, i.e., they quantify local PAC observed between different frequencies of the same signal (within-channel PAC) or two different signals (cross-channel PAC). With the availability of multi-channel EEG data, there is a growing need for methods that can quantify neuronal couplings across the whole brain and across different frequency bands.

Recently, several methods have been introduced to quantify multivariate phase amplitude coupling [10]–[12]. The existing methods are based on matrix factorization and as a result, they model multivariate PAC for a pre-defined pair of frequencies rather than capturing the variation of PAC across space and frequency, simultaneously. For example, phase coupling estimation (PCE) is limited to quantifying CFC between one high frequency and *N* low frequency signals. As such, PCE cannot capture the whole brain CFC dynamics. [11]. Generalized eigendecomposition (gedCFC), on the other hand, is based on generalized eigendecomposition of multi-channel covariance matrices [10]. This is a hypothesis-driven framework and requires *a priori* knowledge about the low and high frequency bands that are coupled with each other. Thus, even though it is a multivariate PAC analysis method, it is limited to two pre-determined frequency bands. In recent work [12], we have introduced a tensor based framework using PARAFAC decomposition to identify multivariate PAC. While this method can capture the multi-way nature of PAC across channels and frequency bands, it is highly dependent on model order selection. Moreover, it identifies the phase and amplitude providing channels separately rather than directly determining the coupled channel pairs. Thus, this method requires additional significance testing of all possible combinations of phase and amplitude providing channels identified through the outer product of the extracted factors.

In this paper, we first compute bivariate PAC values across all phase and amplitude providing channels, frequency bands, and subjects and then represent these pairwise PAC values as a multi-way tensor. We propose a Higher order Robust Principal Component Analysis (HoRPCA) based multivariate framework to capture the spatial and spectral variation of the event-related PAC. HoRPCA decomposes an input tensor into low-rank and spare parts and has been widely used in a variety of outlier detection applications such as distinguishing sparse event-related EEG from the background non-task related brain activity [13]–[15]. It has been previously reported that different behavioral tasks evoke distinct patterns of PAC across the cortex [16]. For example, enhanced PAC in the temporal cortex region is correlated with working memory maintenance tasks [17] whereas an increase in PAC in the medial frontal cortex is related to error processing tasks [18]. During an event-related study, as all subjects perform the same task and respond to the same stimulus, we assume a relatively similar PAC network structure exists for all subjects. A similar assumption was previously made about within frequency synchronization networks [15], [19]. Based on this assumption for event-related activity, HoRPCA based multivariate PAC approach captures the background PAC network in the low-rank part of the tensor while the sparse tensor captures the PAC activity associated with the event of interest. We evaluate the performance of HoRPCA based multivariate PAC approach on synthetic EEG data with varying signal parameters such as noise, subject variability, and volume conduction and compare it with existing multivariate PAC approaches. Finally, we apply the proposed method to EEG recordings collected during a cognitive control study to identify the spatial and spectral dynamics of pairwise PAC associated with different response types and to differentiate between the response types using multivariate analysis of PAC networks.

## II. Background

### A. RID-Rihaczek Time-Frequency Distribution

RID-Rihaczek time-frequency distribution of a signal *x*(*t*) is defined as^1^ [20]:

(1) $$C\left(t,f\right)=\int \int \exp\left(-\frac{{\left(\theta \tau \right)}^{2}}{\sigma }\right)\times \exp\left(j\frac{\theta \tau }{2}\right)A\left(\theta ,\tau \right)e^{-j\left(\theta t+2\pi f\tau \right)}d\tau d\theta ,$$

where $\exp\left(-\frac{{\left(\theta \tau \right)}^{2}}{\sigma }\right)$ corresponds to the Choi-Williams kernel, and $\exp\left(j\frac{\theta \tau }{2}\right)$ corresponds to the kernel function of the Rihaczek distribution [21], *A*(*θ, τ*) refers to the ambiguity function of *x*(*t*) and is defined as:

(2) $$A\left(\theta ,\tau \right)=\int x\left(u+\frac{\tau }{2}\right)x^{*}\left(u-\frac{\tau }{2}\right)e^{j\theta u}du.$$

This is a complex-valued distribution that can be employed to extract the amplitude and phase components of a given signal. In this paper, this time-frequency distribution will be used to extract the phase of the low frequency and amplitude of the high frequency oscillations to compute PAC as detailed in Section III-A.

### B. Tensor Notation

A multidimensional array with *N* modes given as $X\in R^{I_{1}\times I_{2}\times \ldots \times I_{N}}$ is called a tensor, where *x*_*i*_1_,*i*_2_,..*i_N_*_ denotes the (*i*_1_, *i*_2_,..*i_N_*)th element. Column vectors obtained by fixing all indices of the tensor except the one that corresponds to *n*th mode are called mode-*n* fibers. The process of rearranging the tensor elements into a matrix is defined as unfolding or matricization. The mode-*n* unfolding of the tensor X, which is denoted by *X*_(*n*)_, can be obtained by arranging the mode-*n* fibers as the columns of the resulting matrix. The vectorization of X is defined as vec(X).

## III. Methods

### A. Time-Frequency PAC (t-f PAC)

PAC for a given signal is defined as the modulation between the amplitude *A_f_a__* (*t*) of the high frequency (*f_a_*) with the phase *φ_f_p__* (*t*) of the low frequency (*f_p_*) oscillations. The first step in computing PAC between two neuronal oscillations, *x*(*t*) and *y*(*t*) (where the amplitude of *x*(*t*) is coupled with the phase of *y*(*t*)) is to compute the RID-Rihaczek distributions *C_x_* (*t, f*) and *C_y_* (*t, f*) following (1). The amplitude component of *x*(*t*) at the desired frequency *f_a_* is extracted from the frequency constrained time marginal of *C_x_* (*t, f*) as follows [22], [23]:

(3) $$A_{x}^{f_{a}}\left(t\right)=\int_{fa_{1}}^{fa_{2}}C_{x}\left(t,f\right)df,$$

where *f*_*a*_1__ and *f*_*a*_2__ refer to the bandwidth around the high frequency of interest, *f_a_*. The phase component of *y*(*t*) at the desired frequency *f_p_* is computed from the complex RID-Rihaczek distribution *C_y_* (*t, f*) as follows [22], [23]:

(4) $$\phi_{y}^{f_{p}}\left(t\right)=\arg\left[\frac{C_{y}\left(t,f_{p}\right)}{\left|C_{y}\left(t,f_{p}\right)\right|}\right].$$

After quantifying the amplitude and phase components, bivariate PAC between *x*(*t*) and *y*(*t*) can be computed using the amplitude normalized modulation index (MI) proposed by Özkurt and Schnitzler [24] as follows:

(5) $$MI_{x,y}\left(f_{p},f_{a},t\right)=\frac{1}{\sqrt{K}}\frac{\left|\sum_{k=1}^{K}A_{x,k}^{f_{a}}\left(t\right)e^{j\phi_{y,k}^{f_{p}}\left(t\right)}\right|}{\sqrt{{\sum_{k=1}^{K}A_{x,k}^{f_{a}}\left(t\right)}^{2}}},$$

where *K* is the number of trials, $A_{x,k}^{f_{a}}\left(t\right)$ is the extracted amplitude component from *x*(*t*) at high frequency *f_a_* for the *k*th trial and $\phi_{y,k}^{f_{p}}\left(t\right)$ is the extracted phase component at low frequency *f_p_* from *y*(*t*) for the *k*th trial. This metric quantifies PAC as a function of time, low frequency (*f_p_*) and high frequency (*f_a_*) and is between 0 and 1. As time-frequency based phase and amplitude component estimates were used to compute *MI_x, y_*(*f_p_, f_a_, t*), for the remainder of the paper, this PAC index will be referred to as t-f PAC to differentiate it from conventional PAC metrics. MATLAB implementation of this metric is given in [25]. From this t-f PAC, we compute time averaged PAC as: $MI_{x,y}\left(f_{p},f_{a}\right)=\frac{\sum_{t\in T}MI_{x,y}\left(f_{p},f_{a},t\right)}{\left|T\right|}$, where *T* is the time window of interest and |*T*| is the number of time points in that interval. This time averaged PAC, which is constructed by averaging the multi-dimensional t-f PAC estimate within a time window of interest, is used for constructing the PAC networks. As such, although t-f PAC itself is a time-varying metric, the PAC networks are not time-varying.

### B. Statistical Significance Testing

The significance of pairwise PAC values can be determined by surrogate data testing. The surrogate data is generated by following the block swapping procedure suggested in [26]. In this approach, a surrogate amplitude time series was generated by splitting the amplitude time series at a random point and swapping the two obtained time series. This surrogate amplitude time series was then associated with the original phase time series to compute PAC values. This procedure was repeated 100 times to generate 100 random time averaged surrogate PAC values for each frequency pair. Using these values, a threshold value, *Th_i,j_*(*f_p_, f_a_*) is obtained at the 95% confidence interval for each low frequency (*f_p_*) - high frequency (*f_a_*) combination between phase channel *i* and amplitude channel *j*. Time averaged PAC values that surpass this threshold value are considered significant for that low-high frequency pair and used for constructing the PAC network.

We also obtain a threshold $\bar{Th_{i,j}}\left(F_{p},F_{a}\right)$, for all 1 ≤ *i, j* ≤ *N* channel pairs and low and high frequency band pairs by averaging all the threshold values within those frequency bands, i.e., $\bar{Th_{i,j}}\left(F_{p},F_{a}\right)=\frac{\sum_{f_{p}\in F_{p}}\sum_{f_{a}\in F_{a}}Th_{i,j}\left(f_{p},f_{a}\right)}{\left|F_{p}\right|\left|F_{a}\right|}$, where *F_p_* is the low frequency band of interest, *F_a_* is the high frequency band of interest, |*F_p_*| is the number of frequencies in the low frequency band *F_p_* and |*F_a_*| is the number of frequencies in the high frequency band *F_a_*.

### C. PAC Tensor Construction Across Frequency Bands and Subjects

For a channel and frequency band pair, we first compute the pairwise time averaged PAC *MI_i, j_* (*f_p_, f_a_*) for all frequencies of interest, *f_p_* and *f_a_* and compare it with the threshold value *Th_i, j_*(*f_p_, f_a_*). *MI_i, j_* (*f_p_, f_a_*) values that surpass the corresponding threshold values are considered significant and used for computing the average PAC for that channel pair. After quantifying the pairwise time averaged PAC for all EEG channels and across all frequencies, a *N* × *N* weighted and directed connectivity network, *A^F_p_, F_a_^*, is constructed where, $A_{ij}^{F_{p},F_{a}}=\bar{MI_{i,j}}\left(F_{p},F_{a}\right),$ for all 1 ≤ *i, j* ≤ *N*. Here, $\bar{MI_{i,j}}\left(F_{p},F_{a}\right)=\frac{\sum_{f_{p}\in F_{p}}\sum_{f_{a}\in F_{a}}M{I^{*}}_{i,j}\left(f_{p},f_{a}\right)}{\left|F_{p}\right|\left|F_{a}\right|}$, where *F_p_* is the low frequency band of interest, *F_a_* is the high frequency band of interest, |*F_p_*| is the number of frequencies in the low frequency band *F_p_*, |*F_a_*| is the number of frequencies in the high frequency band *F_a_* and

$$MI_{i,j}^{*}\left(f_{p},f_{a}\right)=\{\begin{array}{ll} MI_{i,j}\left(f_{p},f_{a}\right), & \mathrm{if}\;MI_{i,j}\left(f_{p},f_{a}\right)\\ & >Th_{i,j}(f_{p},f_{a}),\\ 0, & \mathrm{otherwise}. \end{array}$$

In this paper, we focus on four different low frequency bands (*F_p_*s), delta (1-3 Hz), theta (4-7 Hz), alpha (8-12 Hz), and beta (13-30 Hz) that may modulate the amplitude of the high frequency (*F_a_*) band gamma (31-100 Hz). After constructing these adjacency matrices for all frequency bands of interest, the goal of multivariate PAC analysis is to detect the spatial and spectral localization of the coupled channel pairs.

Given *A^F_p_,F_a_^*, a 4-way tensor $A\in R^{N\times N\times M\times S}$ is constructed where the first mode corresponds to *N* phase providing channels, second mode corresponds to *N* amplitude providing channels, third mode corresponds to the *M* different low-high frequency band pairs and the fourth mode of the tensor is included to capture the variation of PAC values across *S* subjects.

### D. PARAFAC-Based Multivariate PAC

In this paper, we compare our proposed approach with a previously proposed PARAFAC based multivariate analysis. PARAFAC is a generalization of PCA for multi-way data and has been commonly used to extract information from multi-channel EEG data [27]–[29]. For PARAFAC based multivariate PAC, following [12], a 4-way PARAFAC decomposition is used to express A in terms of its factors across each mode. As A is non-negative, a non-negativity constraint was imposed on the PARAFAC factors as follows:

(6) $$A_{ijkl}=\sum_{r=1}^{R}\lambda_{r}a_{ir}b_{jr}c_{kr}d_{lr,}$$

where, *a_ir_* ≥ 0, *b_jr_* ≥ 0, *c_kr_* ≥ 0 and *d_lr_* ≤ 0 are the elements of the loading vectors across each mode. The first mode factors $a_{r}\in R^{N\times 1}$ provide the spatial loading for the different phase-providing channels whereas $b_{r}\in R^{N\times 1}$ provide the spatial loading for the different amplitude-providing channels. The third mode factors, $c_{r}\in R^{K\times 1}$ correspond to the low frequency bands that modulate the high frequency band. The factors for the fourth mode $d_{r}\in R^{S\times 1}$ contain the signature for each subject. As described in [12], the peaks in the factors of the first two modes allow us to determine the spatial locations of the phase- and amplitude-providing channels while the peaks in the factors of third mode provide the coupled low-high frequency band pairs. In this analysis, the rank, *R* was determined using DIFFerence in FIT (DIFFIT) method proposed in [30] as it was reported to be more suitable for offline analysis [31].

### E. HoRPCA-Based Multivariate PAC

For matrix or two-way data, the limitations of PCA associated with outliers and non-Gaussian errors have been addressed using robust PCA (RPCA) [32]. In this method, a given matrix is decomposed into a low-rank and a sparse matrix. The extension of RPCA to higher-order data, i.e., tensors, has been proposed recently by Goldfarb and Qin [33]. In HoRPCA, the nuclear rank of a matrix is altered by the Tucker rank (Trank) of a tensor. To generalize RPCA to tensors, Trank and *l*_0_ norms are replaced with their convex surrogates CTrank and *l*_1_ norm. Given a tensor, X, the corresponding optimization problem is given as:

(7) $$\min_{L,S}CTrank\left(L\right)+\lambda {\left\|S\right\|}_{1}\;\;\mathrm{subject to}\;\;L+S=X,$$

where L is the low-rank part of the tensor and S is the sparse part of the tensor and ${\left\|S\right\|}_{1}:={\left\|vec\left(S\right)\right\|}_{1}$.

In this paper, we propose to employ HoRPCA [34]–[36] to capture the dynamics of multivariate PAC. HoRPCA is applied to A to obtain the estimates of low-rank component L and sparse component S. The low-rank tensor L accounts for the background or task-independent PAC network whereas the sparse component S represents the task-relevant PAC network.

For computing HoRPCA, we considered the Singleton model proposed in [33], which evaluates the nuclear norm of the tensor as the sum of the nuclear norms of the mode-n unfolding of the tensor, so $CTrank\left(L\right):=\sum_{i=1}^{4}{\left\|L_{\left(i\right)}\right\|}_{*}$. HoRPCA with the Singleton low-rank tensor model can be written as the following convex optimization problem:

(8) $$\min_{L,S}\sum_{i=1}^{4}{\left\|L_{\left(i\right)}\right\|}_{*}+\lambda {\left\|S\right\|}_{1}\;\;\mathrm{subject to}\;\;L+S=A.$$

The low-rank component L and sparse component S can be extracted from A by solving (8) using an alternating direction augmented Lagrangian (ADAL) method [33]. To apply HoRPCA algorithm, we need to tune the regularization parameter *λ*, which controls the trade-off between the sparse and low-rank parts of the tensor A. If *λ* is small, more data points will be allowed to move from L to S, and as *λ* increases fewer data points will be considered anomalous. For this study, following [33], *λ* was set as $\lambda \equiv \frac{1}{\sqrt{I_{\max}}}$ where, $I_{\max}=\max\left(N,N,M,S\right)$.

**Fig. 1.**
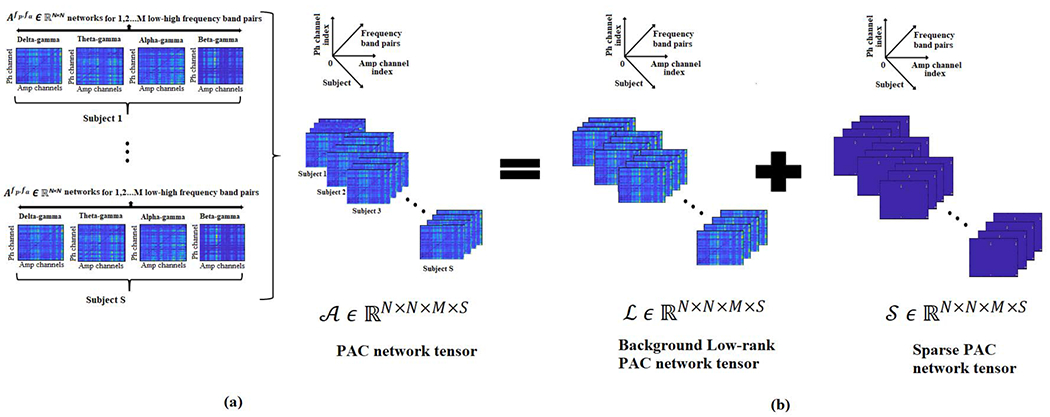
Illustration of the proposed method: **(a)** Generation of the high-order tensor A by concatenating the *N* × *N* PAC networks across M various frequency bands and S subjects. **(b)** Low-rank (L) and sparse tensor (S) decomposition of A through HoRPCA.

For multivariate PAC detection, we are interested in the sparse component S, as the nonzero elements of this tensor will provide us the phase and amplitude providing channels. Each row of S(:,:,m,l) will provide the phase providing channels while each column of S(:,:,m,l) will provide the amplitude providing channels for the *m*th frequency band and *l*th subject. Fig. 1 illustrates the flowchart for the proposed HoRPCA based multivariate PAC detection method. A toy example detailing how the proposed HoRPCA based multivariate t-f PAC measure can detect coupled channel pairs and the frequency band pairs, simultaneously, is provided in the supplementary material.

## IV. Description of Datasets and Experiments

The proposed multivariate PAC approach was validated first on multiple synthesized data sets, and then on EEG data collected during a cognitive control study.

### A. Synthesized Multi-Channel Data

To generate synthesized EEG data, time series data were created at 2, 004 dipole locations in the brain. These dipole locations were based on the standard MRI brain. EEG data were created by considering the fact that the spectrum of EEG data follow the power law, i.e., higher the frequency, weaker the amplitude $\left(P\left(f\right)∼\left(\frac{1}{f^{\beta }}\right)\right)$. The rate of decay of the amplitude is defined by the parameter *β* with *β* = 0, 1, and 2 indicating white noise, pink noise, and Brownian (red) noise, respectively [37]. The frequency spectrum of the human brain has been reported to be similar to Brownian noise [38], [39], hence EEG data was generated from Brownian noise. Time series data from Brownian noise was generated at each of the 2, 004 uncorrelated dipole locations in the brain at a sampling frequency of *F_s_* Hz. Cross-voxel correlations were imposed across all the dipoles by generating a random dipole-to-dipole correlation matrix. The new data matrix was constructed as *Y* = *X^T^V D*^1/2^, where *V* is the eigenvector, *D* is the eigenvalue, and *X* is the data matrix. To project each dipole location to the scalp EEG locations, the forward model was generated by following the openmeeg [40] algorithm implemented in Brainstorm [41].

To introduce phase-amplitude coupling in the simulated data, first the dipole locations corresponding to the high frequency amplitude component and low frequency phase component were chosen. Brownian noise signal *x_B_*(*t*) was generated with a sampling frequency of *F_s_* Hz. Low frequency phase signal *x_f_p__* (*t*) was generated by bandpass filtering *x_B_*(*t*) at the phase providing frequency *f_p_* with a bandwidth of 2 Hz (*f_p_* ± 1 *Hz*). To create the amplitude signal, *x_B_* (*t*) was bandpass filtered at the amplitude providing frequency *f_a_* with a bandwidth of 10 Hz (*f_a_* ± 5 *Hz*) to ensure that the high frequency activity is broadband. PAC was generated using the procedure described by Kramer and Eden [42]. The time locations of relative maxima of the phase signal *x_f_p__* (*t*) were detected. At each maxima, a DC shifted Hanning window with a duration of 42 ms, was multiplied with the amplitude time series. The amplitude signal *x_f_a__* (*t*) with monophasic coupling was generated at high frequency *f_a_* by multiplying the Hanning window with the filtered amplitude time series centered at the relative maxima (peaks) of the phase signal *x_f_p__* (*t*). The coupling intensity is controlled by multiplying the Hanning window itself with a multiplier *I*, where *I* = 1 indicates full coupling and *I* = 0 indicates no coupling. Finally, the time series of the chosen dipole locations were replaced with *x_f_p__* (*t*) for the low frequency and *x_f_a__* (*t*) for the high frequency signal. The whole procedure was repeated *d* times to generate *d* distinctly coupled neuronal oscillations between 2*d* dipole locations (*d* phase providing dipoles and *d* amplitude providing dipoles).

### B. EEG Data

The EEG dataset used to assess the proposed PAC measure was collected during a cognitive control-related error processing study. The experiment conducted was a letter version of the speeded-reaction Flanker task [43]. The study was designed following the experimental protocol and guidelines approved by the Institutional Review Board (IRB) of the Michigan State University (IRB: LEGACY13-144). The data acquisition was performed following the guidelines and regulations established by this protocol. Written and informed consent was collected from each participant before data collection. A string of five letters, which could be congruent (e.g., SSSSS) or incongruent (e.g., SSTSS), was exhibited in front of each participant for each trial. The participants have to select the center letter with a standard mouse with respect to the Flanker letters. The trials began with a 35ms of flanking stimuli (e.g., SS SS). Then, the target stimuli were embedded in the center of the flankers (e.g., SSSSS/SSTSS) and remained for 100 ms (total presentation time is 135ms) followed by an inter-trial interval of varying duration ranging from 1200 to 1700 ms.

Continuous EEG responses were recorded using the ActiveTwo system (BioSemi, Amsterdam, The Netherlands) by placing the 64 Ag–AgCl channels following the international 10/20 system. All EEG signals were sampled at 512 Hz. The trials with artifacts were removed, and volume conduction was minimized using the Current Source Density (CSD) Toolbox [44]. The artifact removed data were considered for analysis in this paper. The trials corresponding to the error and correct responses were separated from each other for analysis. Each trial was one second long. A total of 20 participants were considered for the ongoing study. The inclusion criteria were that the number of trials for error response should be at least 20 or higher. Since the total number of correct/error response trials varies for different participants, the number of trials considered for both responses were kept the same for a fair comparison. The correct response trials were randomly sub-sampled from the whole set of correct responses and the whole procedure was repeated 10 times to select the correct response trials.

### C. Synthesized Data Experiments

The proposed HoRPCA based multivariate PAC method is first tested on the synthesized data set described in Section IV-A. Experiments were conducted to evaluate the robustness of the proposed method against spurious coupling due to noise, subject variability and linear mixing with respect to existing approaches. In these experiments, HoRPCA based method was compared to three other methods, i.e., simple averaging, matrix factorization based gedCFC [10] and PARAFAC based method [12]. For simple averaging method, we first average the delta-gamma, theta-gamma, alpha-gamma and beta-gamma PAC networks for each subject. We also average the thresholds for delta-gamma, theta-gamma, alpha-gamma and beta-gamma networks obtained from the statistical significance testing described in Section III-B for each subject. To detect the coupled channels, we compare the averaged PAC network with the thresholds for each electrode pair. PAC values for gedCFC were computed following the codes provided in [10]. As gedCFC computes PAC between two pre-defined low and high-frequency bands, we compute gedCFC based PAC individually for each frequency band pair combination. PARAFAC based analysis is conducted as described in section III-D. For both gedCFC and PARAFAC based methods, to differentiate the coupled channel pairs from background PAC values, the outputs of these two methods are compared to the average threshold PAC value $\left(\bar{Th_{i,j}}\left(F_{p},F_{a}\right)\right)$ for that channel pair obtained through the statistical significance testing described in Section III-B.

In this section, we conducted two experiments. Experiment 1 focuses on whether the methods can differentiate true coupling from no coupling whereas experiment 2 focuses on the accuracy of the detected channel pairs. The performance evaluation metrics and the description of the experiments are as follows.

#### Performance evaluation metrics:

The performance metrics are:

1. $\mathrm{Precision}=\frac{T\;P}{T\;P+F\;P},$
2. $\mathrm{Recall}/\mathrm{sensitivity}=\frac{T\;P}{T\;P+F\;N},$
3. $\mathrm{F-measure}=\frac{2\times precision\times recall}{precision+recall},$
4. $\mathrm{G-mean}=\sqrt{sensitivity\times specificity},$
  where, $\mathrm{Specificity}=\frac{T\;N}{T\;N+F\;P},$

where

- TP (true positive): coupled pairs detected as coupled.
- FP (false positive): uncoupled pairs detected as coupled.
- FN (false negative): coupled pairs detected as uncoupled.
- TN (true negative): uncoupled pairs detected as uncoupled.

##### 1) Experiment 1: Evaluation of Detection Power of Different Approaches:

In this experiment, we are interested in quantifying the ability of the different methods to differentiate between multi-channel data with and without PAC for different noise levels. This simulation is conducted to ensure that our method is robust against external noise, e.g., measurement or biological noise, and is able to differentiate true coupling from uncoupled oscillatory activity. Two multi-channel EEG datasets were generated. First dataset was generated with theta-gamma coupling (I = 0.7) between any two randomly selected dipole locations. Signals for amplitude and phase providing dipoles were generated following the procedure described in Section IV-A. For all other dipoles, the signals were time series data generated from Brownian noise. This procedure was repeated 20 times to generate a dataset with 20 subjects with different dipole locations and this dataset was referred to as ’dataset with coupling’. For the second dataset, low and high frequency signals were generated at the same dipole locations as the first dataset but the coupling intensity was set to zero (*I* = 0) to generate no-coupling condition. This procedure was also repeated 20 times to generate a dataset with 20 subjects and this dataset was referred to as ’dataset without coupling’. The performance of each approach was determined by evaluating whether each method detected any coupled pairs for each subject or not. Based on this evaluation, detection accuracy in terms of precision, recall, F-measure and G-mean is quantified across subjects for varying signal to noise ratio (SNR) levels (−6dB to 6dB). We repeat the whole experiment 20 times to quantify the variability of each method.

##### 2) Experiment 2: Performance Evaluation With Varying Signal Parameters:

We next conduct experiments assessing the accuracy of the detected coupled channel pairs with respect to various signal parameters, including noise, variability in dipole locations and number of coupled channel pairs. As gedCFC cannot detect multiple channel pairs across multiple frequencies, it was not included in these comparisons. For evaluating the performance of the remaining three methods (HoRPCA, PARAFAC and averaging), for a fair comparison, the number of channel pairs detected by each method was fixed as the actual number of coupled pairs in the data.

#### Robustness to noise:

**Fig. 2.**
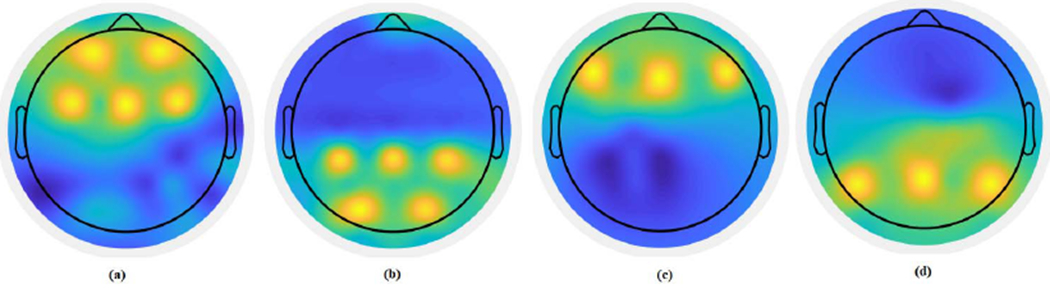
The projections of the dipole locations to the scalp for the phase and amplitude components of the synthesized data for the performance evaluation experiments with varying signal parameters. **(a)** Theta band phase providing dipoles; **(b)** Gamma band amplitude providing dipoles coupled with the theta phase providing dipoles; **(c)** Alpha band phase providing dipoles; **(d)** Gamma band amplitude providing dipoles coupled with the alpha phase providing dipoles.

To evaluate the robustness of the proposed method, we generated synthesized data with additive white Gaussian noise of varying SNR from −6dB to 6dB added to all dipole locations. Assessing the robustness to noise is important as real EEG signals are not simple linear combinations of dipole sources and noise can be generated by both neural and external measurement systems. Eight coupled pairs (5 theta-gamma and 3 alpha-gamma) were generated for each subject between 16 dipole locations (8 phase providing and 8 amplitude providing dipoles) following the steps described in Section IV-A. The projections of these dipole locations are shown in Fig.2. This procedure was repeated ten times to generate a dataset with ten subjects for each SNR level. The whole experiment was repeated 20 times to quantify the variability of each method.

#### Robustness to subject variability:

For multi-subject analysis, methods such as our method which tries to identify coupled channels across subjects, variability across subjects is natural. In this experiment, we evaluate the performance of the proposed method by introducing variability in the location of the coupled channels to determine the robustness of the method against variability across subjects. First, eight multivariate coupled pairs of oscillations (5 theta-gamma and 3 alpha-gamma) were generated for each subject between 16 locations (8 phase providing and 8 amplitude providing) following the steps described in Section IV-A and for the dipoles illustrated in Fig.2. Using this procedure, a dataset was generated with ten subjects. SNR was fixed at 6dB for all subjects. Variability was introduced by changing 5% to 25% of the dipole locations with a step size of 5% for different datasets. We repeated the whole experiment 20 times to quantify the variability of each method.

#### Robustness to volume conduction:

The performance of the proposed measure was also evaluated with increasing number of coupled channel pairs to determine the robustness of the proposed method against volume conduction. Volume conduction is very commonly encountered in EEG and refers to the phenomenon where nearby electrodes are highly correlated with each other due to the conductivity of the scalp [45]. This is important as the number of coupled dipoles increases there will be more interference across channels which may make it harder to detect the truly coupled channels. Synthesized datasets (10 subjects in each dataset with the same number of coupled pairs) were generated by varying the number of coupled channel pairs from 4 to 20 following the steps described in Section IV-A. SNR was fixed at 6dB for all subjects. 75% of the coupled channel pairs were theta-gamma coupled while 25% were alpha-gamma coupled. The whole experiment was repeated 20 times to quantify the variability of each method.

### D. EEG Data Experiments

#### 1) Detection of PAC Networks:

Using the average PAC within a frequency band and time window of interest ($\bar{\mathrm{MI}}$) defined in section III-C, pairwise PAC values were computed across all 64 electrodes for delta-gamma, theta-gamma, alpha-gamma and beta-gamma frequency band combinations for each subject and response type, error and correct. As previous studies indicate increased synchronization associated with the ERN in the time window 0-100 ms [46], the connectivity matrices were constructed by averaging the time-varying PAC within this time window. This resulted in $A_{error}\in R^{64\times 64\times 4\times 20}$ and $A_{correct}\in R^{64\times 64\times 4\times 20}$.

HoRPCA is applied on both $A_{error}\in R^{64\times 64\times 4\times 20}$ and $A_{correct}\in R^{64\times 64\times 4\times 20}$ to obtain the estimates of low-rank components $L_{error}$, $L_{correct}$ and sparse components $S_{error}$, $S_{correct}$. The sparse components indicate that the theta-gamma frequency band pairs have the highest PAC for both EEG error and correct responses. The set of coupled channels for error and correct responses for theta band across all subjects was determined for all 1 ≤ *i, j* ≤ *N* as:

(9) $$\mathrm{Error response}:\;X_{er}\left(i,j\right)=\sum_{i=1}^{20}1_{R^{+}}\left[S_{error}\left(i,j,2,l\right)\right],$$

(10) $$\mathrm{Correct response}:\;X_{cr}\left(i,j\right)=\sum_{l=1}^{20}1_{R^{+}}\left[S_{correct}\left(i,j,2,l\right)\right],$$

$$\mathrm{where},1_{R^{+}}\left[S_{error}\left(i,j,2,l\right)\right]=\{\begin{array}{ll} 1, & \mathrm{if}\;S_{error}\left(i,j,2,l\right)>0,\\ 0, & \mathrm{otherwise}, \end{array}$$

$$\mathrm{and}\;1_{R^{+}}\left[S_{correct}\left(i,j,2,l\right)\right]=\{\begin{array}{ll} 1, & \mathrm{if}\;S_{correct}\left(i,j,2,l\right)>0.\\ 0, & \mathrm{otherwise}. \end{array}$$

The detected channel combinations for error (*X_er_*) and correct (*X_cr_*) responses are referred to as PAC networks for error and correct responses, respectively.

#### 2) Classification Based on Detected PAC Network:

A classification experiment was conducted to determine if the detected multivariate PAC networks can be used as features to discriminate between error and correct responses. As the proposed method uses the sparse part of the tensor for classification and since this sparse part is data dependent, the classification was performed by following the sparse representation classification (SRC) approach described in [47], [48].

We first generate training and test samples from $A_{error}\in R^{64\times 64\times 4\times 20}$ and $A_{correct}\in R^{64\times 64\times 4\times 20}$ using 5-fold cross-validation. For each validation, we take *I* subjects (both error and correct responses) to generate a test set $A_{test}\in R^{64\times 64\times 4\times 2I}=\left[A_{err_{test}},A_{crr_{test}}\right]$. We then compute HoRPCA for the remaining *J* = 20 − *I* subjects in the training set separately for error $A_{err_{train}}\in R^{64\times 64\times 4\times J}$ and correct responses $A_{crr_{train}}\in R^{64\times 64\times 4\times J}$ to obtain two separate training feature matrices corresponding to error and correct responses. The feature matrices are the fourth mode slices of the sparse tensors $S_{err_{train}}\in R^{64\times 64\times 4\times J}$ and $S_{crr_{train}}\in R^{64\times 64\times 4\times J}$ corresponding to each subject *j* ∈ {1, 2, …, *J*} in the training set. Next, for each subject and response type in the test set $\left(Y_{k}=A_{\mathrm{test}}(:,:,:,k)\right)$, we concatenate the error and correct tensors with the PAC tensor corresponding to each test sample $\left(Y_{k}\right)$ separately, i.e., we end up with two tensors $\left[A_{err_{train}},Y_{k}\right]\in R^{64\times 64\times 4\times (J+1})$ and $\left[A_{crr_{train}},Y_{k}\right]\in R^{64\times 64\times 4\times (J+1})$. We perform HoRPCA on each of these tensors and extract the sparse matrix corresponding to the test sample. This results in two feature matrices for our test sample, one based on projection with respect to the error training samples and the other based on projection with respect to correct training samples. For each of these feature matrices, we compute the distances between the feature matrix from the test sample and the feature matrices obtained from the training data. The test sample is assigned to the class to which it has the smallest distance, i.e., Nearest Neighbor (NN) classification. We repeat this procedure across all test samples and multiple 5-folds and report the average accuracy. The overall procedure is summarized in Algorithm 1.

**Algorithm 1:** Sparse Representation-Based Classification (SRC) of PAC Networks

	**Input** : Training tensors: $A_{err_{train}}\in R^{64\times 64\times 4\times J}$ and $A_{crr_{train}}\in R^{64\times 64\times 4\times J}$; Test tensor: $A_{test}\in R^{64\times 64\times 4\times 2I}=\left[A_{err_{test}},A_{crr_{test}}\right],$ where, $A_{err_{test}}\in R^{64\times 64\times 4\times I},A_{crr_{test}}\in R^{64\times 64\times 4\times I}.$
	**Output**: Label of test samples in $A_{test}.$
**1**	Perform HoRPCA on $A_{err_{train}}$ and $A_{crr_{train}}$ using (8) to obtain training features $S_{err_{train}}$ and $S_{crr_{train}}$ .
**2**	**for** *k* = 1 *to* 2*I* **do**
**3**	$Y_{k}=A_{test}\left(:,:,:,k\right).$
**4**	Using (8), perform HoRPCA on $A_{err_{train}}^{*}=\left[A_{err_{train}},Y_{k}\right],A_{crr_{train}}^{*}=\left[A_{crr_{train}},Y_{k}\right]$ to obtain $S_{err_{train}}^{*}$ and $S_{crr_{train}}^{*}$.
**5**	$S_{err_{test}}^{k}\leftarrow S_{err_{train}}^{*}\left(:,:,2,J+1\right)$
**6**	$S_{crr_{test}}^{k}\leftarrow S_{crr_{train}}^{*}\left(:,:,2,J+1\right).$
**7**	**for** *l* = 1 *to J* **do**
**8**	$d_{k,err}^{l}={\left\|S_{err_{train}}\left(:,:,2,l\right)-S_{err_{test}}^{k}\right\|}_{2}$
**9**	$d_{k,crr}^{l}={\left\|S_{crr_{train}}\left(:,:,2,l\right)-S_{crr_{test}}^{k}\right\|}_{2}$
**10**	**end**
**11**	$\mathrm{Label}\left(Y_{k}\right)=\arg\min_{err,crr}\left(d_{k,err}^{l},d_{k,err}^{l}\right)$ for all *l*.
**12**	**end**

We compare the classification performance of HoRPCA with simple averaging, gedCFC and PARAFAC based methods. As simple averaging and gedCFC methods are not data dependent, to classify the error and correct responses for these two methods, we employ simple NN classifier without the SRC based algorithm. For simple averaging method, the classification was performed using 5-fold cross-validation with an NN classifier. The feature matrices in this case are the 64 × 64 PAC networks averaged across frequency bands corresponding to each subject and response type. The classification for gedCFC was also performed by using 5-fold cross-validation with NN classifier. In this case, the features are the multivariate PAC values for the theta-gamma band extracted by gedCFC. For PARAFAC based method, as it is also data dependent similar to HoRPCA, the classification was performed following the SRC based NN-classifier approach described in Algorithm 1. For all cases, the performance was evaluated in terms of precision, recall, F-measure and G-mean.

## V. Results

### A. Synthesized Data Results

#### 1) Results for Experiment 1:

The evaluation performance of the detection power for the four methods for various SNR values is reported in Table I. From Table I, it can be seen that the proposed HoRPCA based PAC approach outperforms gedCFC in all cases and can detect the presence of PAC even for highly noisy data (−6dB) with a F-measure close to 0.85. The detection power of PARAFAC based approach was also high and comparable to the proposed HoRPCA based approach. The high performance exhibited by the HoRPCA and PARAFAC based methods indicates that the tensor based multi-way analysis takes full advantage of the multi-linear structure of the data, which improves the classification performance compared to matrix factorization-based methods like gedCFC. Averaging the networks has the worst performance (i.e., performance around the chance level) for all SNR levels indicating loss of information due to averaging.

#### 2) Results for Experiment 2:

The performance of the three approaches for various SNR levels, variability in dipole locations and number of coupled channel pairs are depicted in Tables II, III and IV, respectively. From Tables II, III and IV, we can see that the proposed HoRPCA based method outperforms PARAFAC and the averaging based methods in all cases. HoRPCA detected the coupled channels correctly with more than 80% precision and recall even for high noise levels, e.g. −6dB, (Table II). HoRPCA is also shown to be more robust against subject variability (Table III). It is also less sensitive to the number of coupled channel pairs (Table IV). While PARAFAC based multivariate PAC detection performs well for low noise levels and less variability in the data, its performance deteriorates quickly as the uncertainty in the data increases. For example, PARAFAC is unable to detect the correct coupled channel pairs in the presence of variability across subjects. This can be explained by the fact that PARAFAC based method detects the channel pairs which are common across all subjects. In all cases, averaging the networks yielded the worst performance and was found to be more sensitive to different signal parameters. This is due to the fact that averaging cannot capture variability across subjects and frequency bands. The standard deviations reported in these Tables reflect that the variability of the HoRPCA method is smaller than others across multiple realizations of the experiments. This indicates the stability of the proposed method.

### B. EEG Data Results

#### 1) PAC Network Detection Result:

**Fig. 3.**
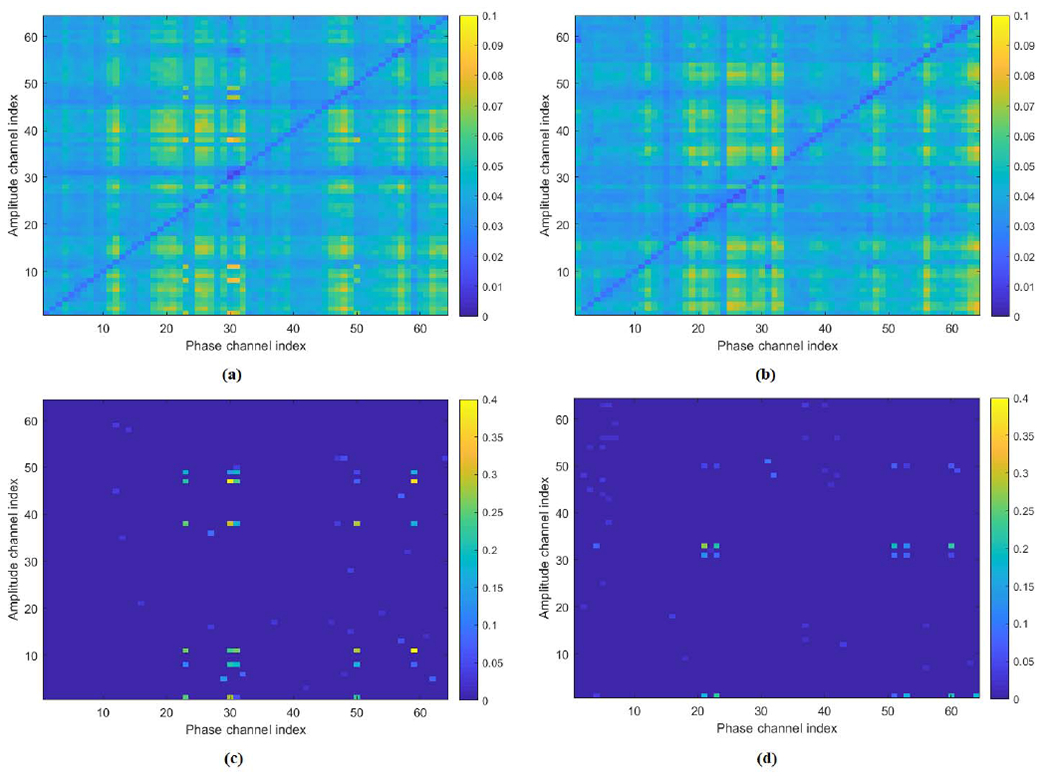
Low-rank and sparse networks extracted for the error and correct responses averaged across subjects. **(a)** Average low-rank PAC network for error response; **(b)** Average low-rank PAC network for correct response; **(c)** Average sparse PAC network for error response; **(d)** Average sparse PAC network for correct response.

The low-rank and sparse networks extracted for the theta band of error and correct responses averaged across subjects is shown in Fig.3. This figure shows that the low-rank parts of the tensor for both response types are similar, while the sparse parts illustrate the differences in the coupled brain regions for the two response types.

**Fig. 4.**
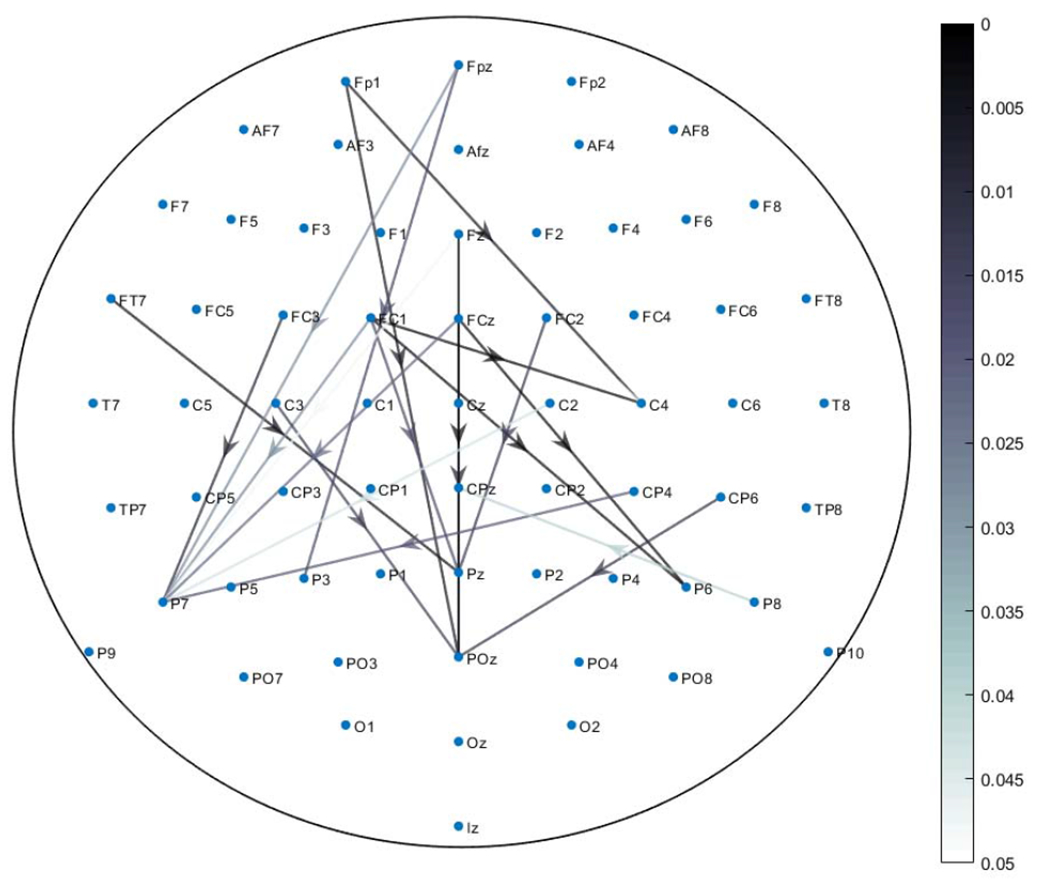
PAC network of *p*-values showing significant difference between error and correct responses. The color scale from white to black indicates the level of significance (Wilcoxon Signed Rank Sum Test with *p* < 0.05). The arrows originate from phase providing channel.

This observation is also verified by statistical testing. A Wilcoxon Signed Rank Sum Test (*p* < 0.05) with Bonferroni correction is conducted to determine the statistical significance of the difference between ERN and CRN PAC networks across subjects. The statistical testing revealed no significant difference between the low rank networks but significant difference was observed between sparse networks. We constructed a *p*-value PAC network that shows the channel pairs with significant difference between the sparse networks corresponding to error and correct responses. The *p*-value PAC network is depicted in Fig.4.

**Fig. 5.**
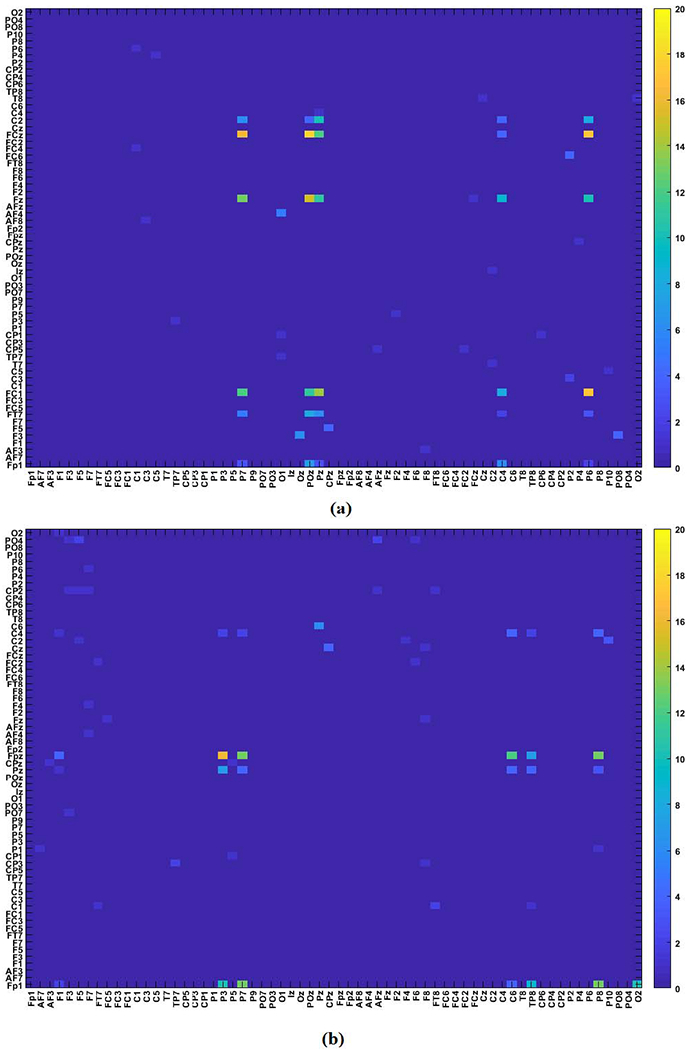
The detected channel combinations across all subjects for **(a)** error response (*X_er_*); and **(b)** correct response (*X_cr_*) for theta frequency band.

From the extracted sparse networks, we detected the channel combinations for error (*X_er_*) and correct (*X_cr_*) responses as shown in Fig.5 (a) and (b), respectively. The colormap shows the number of subjects for which a channel combination was detected. The yellow pixels indicate channel pairs with coupling across the majority of subjects, while the light blue pixels correspond to channel pairs with coupling for only a few number of subjects.

**Fig. 6.**
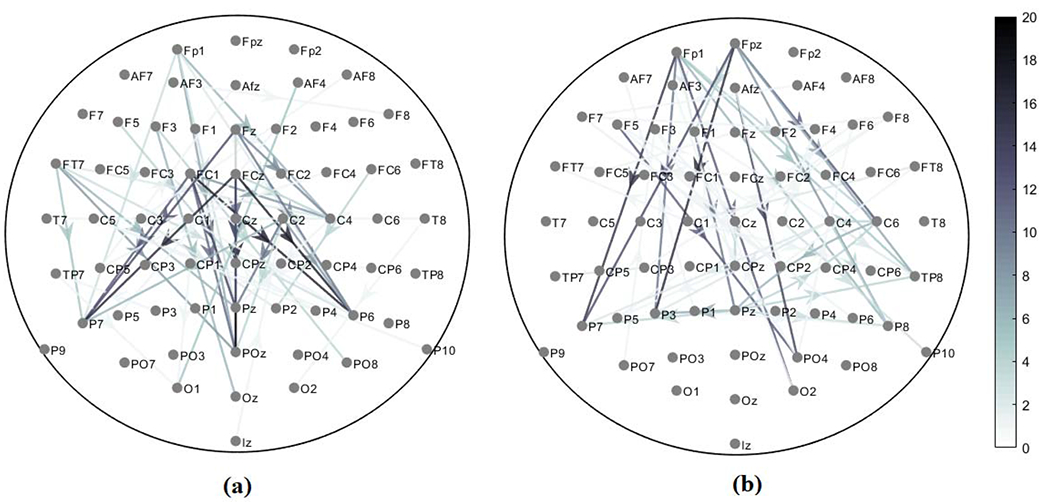
Theta-gamma PAC networks detected for **(a)** Error response and **(b)** Correct response. The color scale from white to black indicates the number of subjects for which a channel pair was detected. The arrows originate from phase providing channel.

The channel pairs (phase-amplitude coupled channels) detected for a majority of subjects for error response are: FP1-POz, FP1-Pz, FP1-C4, F3-Oz, FT7-POz, FC1-P7, FC1-POz, FC1-Pz, FC1-C4, FC1-P6, Fz-C4, Fz-P6, FCz-P7, FCz-POz, FCz-Pz, FCz-P6, C2-Pz, and C2-P6. Similarly, for correct response the channel pairs (phase-amplitude coupled channels) detected for a majority of subjects are: FP1-P7, FP1-P3, FP1-O2, F1-Pz, F5-PO4, FPz-P7, FPz-P3, FPz-P8, FPz-C6, FPz-TP8, AFz-PO4 and C6-Pz. The resulting theta-gamma band PAC networks for error and correct responses are shown in Fig.6(a) and (b) respectively.

#### 2) Classification Experiment Result:

Table V shows the comparison of classification performance for 50 repetitions of the 5-fold cross-validation in terms of precision, recall, F-measure and G-mean. From this Table, it can be seen that the proposed HoRPCA based multivariate method results in very high accuracy (F-measure and G-mean > 0.98) compared to averaging (F-measure and G-mean close to 0.70) and matrix factorization-based gedCFC (F-measure and G-mean close to 0.85). The discrimination power of PARAFAC was slightly lower than the proposed method. The increased discrimination power of the tensor based methods indicates that the tensor based multivariate t-f PAC approaches are more effective at identifying the brain regions central to PAC.

## VI. Discussion

In this paper, a HoRPCA based approach was proposed to identify the spatial and spectral components of multivariate PAC across all channels, frequency bands, and subjects. As illustrated in Fig.5, HoRPCA can determine the coupled channel pairs directly from the row and column indices of the non-zero elements of the sparse tensor component for a given frequency band. This approach provides certain advantages over PARAFAC decomposition of the same tensor. First, with the PARAFAC model, order selection is an open problem. While the first factor across each mode can be used to identify the coupled channel pairs and frequency bands, these factors are only effective at capturing the largest variance in the data and are not necessarily robust to outliers and variations in the data such as the ones considered in Tables II, III and IV. Second, PARAFAC identifies the amplitude and phase providing channels individually rather than identifying the actual channel pairs. This results in *P* × *Q* possible channel pairs, where *P* is the number of amplitude providing channels and *Q* is the number of phase providing channels. In order to determine the actual coupled channel pairs, one needs to do significance testing which is computationally expensive. Estimation of coupled channel pairs from the indices of the sparse matrix in the HoRPCA method overcomes these limitations. Based on the F-measure values reported for various simulations, HoRPCA based method outperforms PARAFAC and averaging based method and is more robust to varying signal conditions such as noise level, subject variability and number of couplings. HoRPCA is also more robust to noise compared to gedCFC indicating the advantage of tensor representation with respect to matrix factorization based methods (Table I).

In the analysis of EEG data, the proposed approach detected event-related theta-gamma PAC during error and correct responses with higher PAC for error response. This finding is consistent with prior studies where theta-gamma coupling was reported during visual tasks like working memory processing and serial memory recall [9]. Theta-gamma PAC was also reported in an error processing MEG study [18] and in error/correct trial learning [49]. A possible explanation for the presence of higher theta-gamma PAC during error response is that error trials may reflect a miscoding of information, which leads to a large-scale functional integration across theta-gamma frequency bands to improve the performance after an error response [49].

Cross-frequency networks extracted through HoRPCA are sparse networks as the background couplings are discarded (Fig.3, Fig.6). Analysis of the low-rank parts of the tensors for both error and correct responses showed that there was no significant difference between the two response types based on solely the low-rank tensor (Fig.3). This observation justifies our hypothesis that the low-rank part of the tensor captures background PAC activity while the sparse part corresponds to response-evoked PAC. Observation of the detected PAC networks shows that the networks corresponding to the correct response were concentrated between frontal theta phase providing channels and parietal gamma amplitude channels. On the other hand, networks for error response were concentrated between frontal-central theta phase activity and parietal gamma activity. Comparison of ERN and CRN networks through statistical significant testing revealed that the difference was centered around the medial frontal cortex, which has been previously reported to be active during error processing [18]. Prior studies also hypothesized that error-related negativity initiates the medial frontal based top-down control mechanisms to improve the performance after an error response [50], [51]. Thus, the PAC networks extracted by the proposed HoRPCA approach are consistent with previous literature reflecting higher theta-gamma coupling in the medial frontal cortex and relating this with error-related negativity. The high classification accuracy achieved by HoRPCA based multivariate PAC also indicates that multivariate PAC can be successfully employed to characterize cross-frequency connectivity networks associated with error and correct responses.

Although the proposed multivariate approach is promising, there are some limitations. First, the multivariate analysis was conducted by constructing the network with amplitude extracted only from the gamma frequency band. The low-high frequency band combinations were chosen based on the literature indicating the presence of PAC mainly between the low frequency phase (typically 5–12 Hz) and high frequency amplitude (typically 30–100Hz) [9]. However, other low-high frequency combinations such as delta-theta/alpha/beta, theta-alpha/beta and alpha-beta can also be studied by adding those networks in A. Another limitation is that the multivariate measure focuses on the average PAC values across a pre-determined time window. However, there is growing empirical evidence that PAC is dynamic, varying across time. Consequently, future work could include extending the proposed measure to capture the temporal and spatial dynamics of PAC.

## VII. Conclusion

In this study, we proposed a HoRPCA based multivariate PAC framework to capture the dynamics of cross-frequency PAC across various brain regions and frequencies. The empirical results obtained from the analyses of simulated and EEG data supported our hypothesis that the sparse tensor extracted through HoRPCA can capture the spectral and spatial dynamics of event-related multivariate PAC network while discarding the background perturbations as part of the low-rank tensor. With these unique properties, the proposed HoRPCA based multivariate approach can lead to obtaining a complete understanding of connectivity across frequency bands and brain regions.

## Supplementary Material

supp1-3092890

## Figures and Tables

**TABLE I:** Comparison of PAC Detection Power Obtained With HoRPCA, gedCFC, PARAFAC and Averaging Based Methods for Different SNR Values. The table depicts the Mean±Standard Deviation of Evaluation Matrices for 20 Repetition of Each Experiment

SNR (dB)	Multivariate PAC with Averaging					gedCFC based multivariate PAC					PARFAC based multivariate PAC					HoRPCA based multivariate PAC			
	Precision	Recall	F-measure	G-mean		Precision	Recall	F-measure	G-mean		Precision	Recall	F-measure	G-mean		Precision	Recall	F-measure	G-mean
−6dB	0.366±0.1112	0.384±0.1143	0.375±0.1127	0.359±0.1145		0.747±0.0829	0.784±0.0921	0.765±0.0905	0.759±0.0922		0.797±0.0765	0.837±0.0791	0.817±0.0778	0.812±0.0793		0.810±0.0674	0.891±0.0647	**0.849±0.0630**	**0.840±0.0617**
−3dB	0.432±0.1127	0.480±0.1097	0.457±0.1110	0.429±0.1128		0.793±0.0878	0.833±0.0805	0.813±0.0811	0.808±0.0807		0.844±0.0732	0.887±0.0727	0.865±0.0726	0.862±0.0704		0.855±0.0603	0.941±0.0620	**0.896±0.0594**	**0.890±0.0586**
0dB	0.505±0.1001	0.500±0.1030	0.492±0.1014	0.503±0.1031		0.840±0.0780	0.882±0.0808	0.860±0.0794	0.857±0.0810		0.891±0.0630	0.936±0.0628	0.913±0.0626	0.911±0.0628		0.918±0.0618	0.973±0.0506	**0.945±0.0522**	**0.942±0.0518**
3dB	0.585±0.0904	0.556±0.0933	0.570±0.0919	0.580±0.0936		0.914±0.0678	0.952±0.0530	0.936±0.0591	0.934±0.0545		0.985±0.0502	0.952±0.0427	0.974±0.0429	0.977±0.0427		0.962±0.0421	0.987±0.0415	**0.978±0.0402**	**0.981±0.0337**
6dB	0.617±0.0828	0.556±0.0928	0.585±0.0912	0.604±0.0832		0.936±0.0652	0.963±0.0599	0.949±0.0585	0.947±0.0531		1.000±0.0000	1.000±0.0000	**1.000±0.0000**	**1.000±0.0000**		1.000±0.0000	1.000±0.0000	**1.000±0.0000**	**1.000±0.0000**

**TABLE II:** Comparison of Detected Channel Pair Accuracy for Various SNRs Obtained With HoRPCA, PARAFAC and Averaging Based Methods. The Table Depicts the Mean±Standard Deviation of Evaluation Matrices for 20 Repetition of Each Experiment

SNR (dB)	Multivariate PAC with Averaging					PARAFAC based multivariate PAC					HoRPCA based multivariate PAC			
	Precision	Recall	F-measure	G-mean		Precision	Recall	F-measure	G-mean		Precision	Recall	F-measure	G-mean
−6dB	0.380±0.1029	0.380±0.1029	0.380±0.1029	0.611±0.0807		0.704±0.0925	0.704±0.0925	0.704±0.0925	0.837±0.0719		0.818±0.0715	0.818±0.0715	**0.818±0.0715**	**0.902±0.0541**
−3dB	0.479±0.0990	0.479±0.0990	0.479±0.0990	0.690±0.0768		0.794±0.0564	0.794±0.0564	0.794±0.0564	0.883±0.0479		0.843±0.0461	0.843±0.0461	**0.843±0.0461**	**0.916±0.0240**
0dB	0.549±0.0928	0.549±0.0928	0.549±0.0928	0.739±0.0568		0.836±0.0563	0.836±0.0563	0.836±0.0663	0.902±0.0396		0.902±0.0310	0.902±0.0310	**0.902±0.0310**	**0.949±0.0197**
3dB	0.608±0.0764	0.608±0.0764	0.608±0.0764	0.778±0.0479		0.919±0.0540	0.919±0.0540	0.919±0.0540	0.958±0.0282		0.949±0.0289	0.949±0.0289	**0.949±0.0289**	**0.971±0.0150**
6dB	0.663±0.0859	0.633±0.0859	0.663±0.0859	0.813±0.0544		0.968±0.0179	0.968±0.0179	0.968±0.0179	0.984±0.0091		0.999±0.0038	0.999±0.0038	**0.999±0.0019**	**0.999±0.0038**

**TABLE III:** Comparison of Detected Channel Pair Accuracy for Variability in Channel Locations Obtained With HoRPCA, PARAFAC and Averaging Based Methods. The Table Depicts the Mean±Standard Deviation of Evaluation Matrices for 20 Repetition of Each Experiment

Variability (%)	Multivariate PAC with Averaging					PARFAC based multivariate PAC					HoRPCA based multivariate PAC			
	Precision	Recall	F-measure	G-mean		Precision	Recall	F-measure	G-mean		Precision	Recall	F-measure	G-mean
0%	0.663±0.0859	0.663±0.0859	0.663±0.0859	0.813±0.0544		0.968±0.0179	0.968±0.0179	0.968±0.0179	0.984±0.0091		0.999±0.0038	0.999±0.0038	**0.999±0.0038**	**0.999±0.0019**
5%	0.609±0.0758	0.609±0.0758	0.609±0.0758	0.778±0.0624		0.903±0.0292	0.903±0.0292	0.903±0.0292	0.950±0.0155		0.979±0.0112	0.979±0.0112	**0.979±0.0112**	**0.989±0.0056**
10%	0.561±0.0842	0.561±0.0842	0.561±0.0842	0.747±0.0631		0.820±0.0377	0.820±0.0377	0.820±0.0377	0.905±0.0225		0.950±0.0256	0.950±0.0256	**0.950±0.0256**	**0.975±0.0132**
15%	0.443±0.0903	0.443±0.0903	0.443±0.0903	0.662±0.0630		0.770±0.0472	0.770±0.0472	0.770±0.0472	0.877±0.0248		0.925±0.0284	0.925±0.0284	**0.925±0.0284**	**0.962±0.0155**
20%	0.400±0.0971	0.400±0.0971	0.400±0.0971	0.632±0.0654		0.721±0.0549	0.721±0.0549	0.721±0.0549	0.848±0.0338		0.906±0.0321	0.906±0.0321	**0.906±0.0321**	**0.951±0.0174**
25%	0.338±0.1046	0.338±0.1046	0.338±0.1046	0.581±0.0666		0.684±0.0701	0.684±0.0701	0.684±0.0701	0.826±0.0432		0.861±0.0444	0.861±0.0444	**0.861±0.0444**	**0.928±0.0236**

**TABLE IV:** Comparison of Detected Channel Pair Accuracy for Various Number of Coupled Channel Pairs Obtained With HoRPCA, PARAFAC and Averaging Based Methods. The Table Depicts the Mean±Standard Deviation of Evaluation Matrices for 20 Repetition of Each Experiment

Coupled Pairs	Multivariate PAC with Averaging					PARAFAC based multivariate PAC					HoRPCA based multivariate PAC			
	Precision	Recall	F-measure	G-mean		Precision	Recall	F-measure	G-mean		Precision	Recall	F-measure	G-mean
4	0.724±0.0917	0.724±0.0917	0.724±0.0917	0.849±0.00619		1.000±0.0000	1.000±0.0000	**1.000±0.0000**	**1.000±0.0000**		1.000±0.0000	1.000±0.0000	**1.000±0.0000**	**1.000±0.0000**
8	0.663±0.0859	0.663±0.0859	0.663±0.0859	0.813±0.0544		0.968±0.0179	0.968±0.0179	0.968±0.0179	0.984±0.0091		0.999±0.0038	0.999±0.0038	**0.999±0.0038**	**0.999±0.0019**
12	0.564±0.0869	0.564±0.0869	0.564±0.0869	0.748±0.0592		0.909±0.0408	0.909±0.0408	0.909±0.0408	0.953±0.0217		0.961±0.0198	0.961±0.0198	**0.961±0.0198**	**0.980±0.0103**
16	0.496±0.0927	0.496±0.0927	0.496±0.0927	0.700±0.0653		0.858±0.0535	0.858±0.0535	0.858±0.0535	0.925±0.0395		0.927±0.0267	0.927±0.0267	**0.927±0.0267**	**0.963±0.0139**
20	0.473±0.1032	0.473±0.1032	0.473±0.1032	0.685±0.0701		0.808±0.0763	0.808±0.0763	0.808±0.0763	0.897±0.0531		0.878±0.0565	0.878±0.0565	**0.878±0.0565**	**0.937±0.0302**

**TABLE V:** Classification of EEG Error and Correct Responses Based Multivariate PAC Networks Computed Using Averaging, gedCFC, PARAFAC and HoRPCA Based Methods

Method	Precision	Recall	F-measure	G-mean
Averaging	0.685±0.243	0.726±0.230	0.678±0.199	0.701±0.163
gedCFC	0.801±0.178	0.915±0.137	0.833±0.115	0.842±0.110
PARAFAC	0.906±0.141	**1.000±0.00**	0.945±0.0879	0.946±0.0797
HoRPCA	**0.975±0.061**	**1.000±0.00**	**0.986±0.033**	**0.984±0.038**

## Footnotes

1 All integrals are from −∞ to ∞ unless otherwise noted.
