# Supplementary material for: Multivariate Analysis of Bivariate Phase-Amplitude Coupling in EEG Data Using Tensor Robust PCA
Source: IEEE Trans Neural Syst Rehabil Eng. Author manuscript; Available in PMC 2021 Oct 25. (PMC8544646; doi:10.1109/TNSRE.2021.3092890)
Supplement: supp1-3092890 [file NIHMS1723808-supplement-supp1-3092890.pdf]

Supplemental Material

Tamanna T. K. Munia, *Member, IEEE*, and Selin Aviyente, *Senior Member, IEEE*

I. A Toy Example of Proposed HoRPCA based PAC Measure

This experiment was conducted to illustrate how the proposed HoRPCA based multivariate t-f PAC measure can detect coupled channel pairs and the frequency band pairs, simultaneously. For this experiment, two low frequency phase components were generated, one in the fronto-central region at 5 Hz (theta frequency band) and another in the pre-frontal/dorsolateral region at 10 Hz (alpha frequency band). Two amplitude providing components were generated at the occipital cortex, one at 60 Hz with its amplitude modulated by the phase of theta and another at 68 Hz with its amplitude modulated by the phase of alpha band oscillations. This procedure was repeated 10 times to correspond to 10 subjects. The coupling strength between theta-gamma and alpha-gamma were selected to be the same at 0.8. The projections of the dipole locations are shown in Fig. 1.

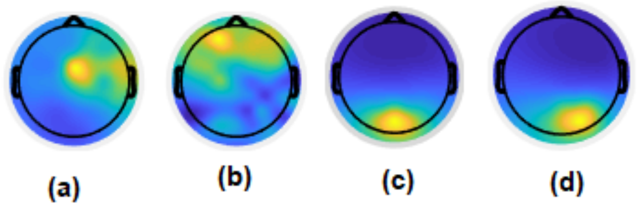

Fig. 1. The projections of the dipole locations to the scalp for the phase and amplitude components of the synthesized data for the multivariate PAC detection experiment. (a) Theta band (5 Hz) phase providing dipole; (b) Alpha band (10 Hz) phase providing dipole; (c) Gamma band (60 Hz) amplitude providing dipole; (d) Gamma band (68 Hz) amplitude providing dipole.

t-f PAC values for all possible channel combinations ($N = 64$) were computed and the 64×64 PAC connectivity networks were generated for delta-gamma, theta-gamma, alpha-gamma and beta-gamma frequency band pairs for all 10 subjects yielding $\mathcal{A} \in \mathbb{R}^{64\times64\times4\times10}$. HoRPCA is applied on $\mathcal{A} \in \mathbb{R}^{64\times64\times4\times10}$ to obtain the estimates of low-rank component $\mathcal{L}$ and sparse component $\mathcal{S}$. The extracted $\mathcal{L}$ and $\mathcal{S}$ along with $\mathcal{A}$ for all four low-high frequency band pairs averaged over the 10 synthesized subjects are shown in Fig.2.

From Fig.2 (c), we can see that the sparse matrix gives us the PAC phase providing channel index 47 (FCz) and amplitude providing channel index 30 (POz) for theta band along with phase providing channel index 3 (AF3) and amplitude providing channel index 59 (P6) for alpha frequency band. No coupled channel pairs were found for delta and gamma frequency bands as there was no PAC in these two frequency bands. Thus, the proposed measure can accurately detect multivariate PAC by detecting the coupled channel pairs and the respective frequency band pairs, simultaneously.

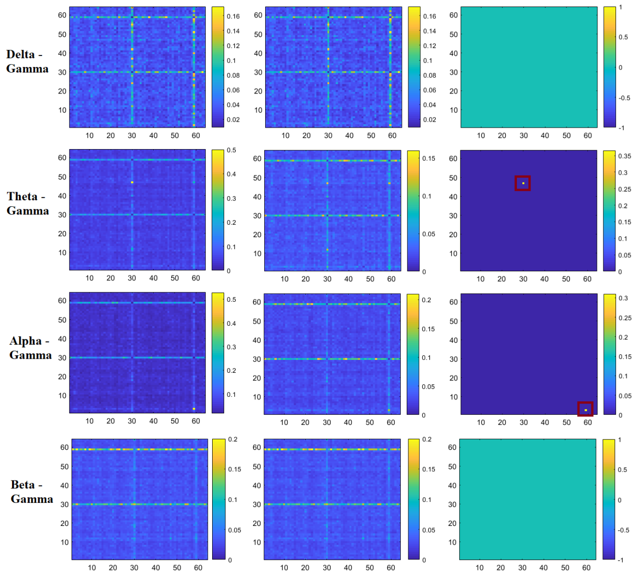

Fig. 2. (a) Multivariate PAC matrices; (b) the low-rank parts and (c) the sparse parts for delta (first row), theta (second row), alpha (third row) and beta (fourth row) bands averaged over 10 subjects of $\mathcal{A} \in \mathbb{R}^{64\times64\times4\times10}$. Detected channel pairs with multivariate PAC are marked with red squares.
